# Impact of Rehabilitation on Time to Home Discharge after Hip Fracture Surgery: A Retrospective Observational Study Using the Japanese Nationwide Database of Diagnosis Procedure Combination

**DOI:** 10.1298/ptr.25-E10360

**Published:** 2026-02-20

**Authors:** Daiki KATO, Kunio TARASAWA, Koki ABE, Kiyohide FUSHIMI, Kenji FUJIMORI

**Affiliations:** 1 Department of Health Administration and Policy, Tohoku University Graduate School of Medicine, Japan; 2 Department of Health Policy and Informatics, Institute of Science Tokyo, Japan

**Keywords:** Diagnosis procedure combination, Home discharge, Hip fracture, Length of stay, Rehabilitation

## Abstract

**Objectives:**

The impact of rehabilitation (RH) started on postoperative day 0 (Day 0) and the association between intensive RH and time to home discharge after hip fracture surgery remain unclear. We aimed to investigate the influence of the RH starting date and RH provision volume on time to home discharge.

**Methods:**

Using the Diagnosis Procedure Combination database, we retrospectively analyzed patients who underwent surgery within 7 days of admission between April 2020 and March 2022. RH starting date was classified as Day 0, postoperative day 1 (Day 1), postoperative day 2 (Day 2), or postoperative day 3 or later (≥Day 3). RH provision volume was calculated as the average number of daily RH units. Time to home discharge was used as the outcome, with home discharge as the event occurrence. Cox proportional hazards regression models were used to examine these associations.

**Results:**

A total of 207450 patients were included. Both variables had a statistically significant association with time to home discharge. The adjusted hazard ratio based on RH started on ≥Day 3 was 0.97 (95% confidence interval: 0.93–1.02) for Day 2, 1.05 (1.01–1.09) for Day 1, and 1.18 (1.10–1.27) for Day 0. The adjusted hazard ratio based on the lowest dose (0–1.0 units/day) was 1.12 (1.08–1.15) for 1.1–2.0 units/day, 1.10 (1.06–1.14) for 2.1–3.0 units/day, 1.04 (1.00–1.08) for 3.1–4.0 units/day, 1.09 (1.04–1.14) for 4.1–5.0 units/day, and 1.11 (1.06–1.17) for ≥5.1 units/day.

**Conclusions:**

Starting RH on Day 0 was suggested to potentially facilitate faster or easier home discharge. Additionally, providing at least one unit of RH appears effective.

## Introduction

Hip fractures are a significant public health concern. In Japan, approximately 200000 hip fractures were reported in 2017^1)^. In the United States, the number is expected to double from 250000 in 1990 to 500000 by 2040^2)^. This number is increasing worldwide and is estimated to grow to approximately 7 million cases worldwide by 2050^3)^. Furthermore, these fractures not only affect the patient’s ability to perform activities of daily living (ADL)^4)^ but also have an impact on long-term prognosis, decreased quality of life^5)^, and increased mortality^6)^. As a result, hip fractures are often associated with several problems, and it is essential to provide optimal care to these patients. Rehabilitation (RH) is one of these measures. However, further validation is needed to determine how effective postoperative RH is for these patients.

Early RH is generally recommended in guidelines around the world^7–9)^. A past study reported that starting physical therapy on postoperative day 0 (Day 0) significantly reduced the length of stay by 0.21 days compared to starting physical therapy on the first day after surgery (Day 1)^10)^. However, there are currently few related papers on this topic. In addition, the volume of RH provision remains controversial. Several studies have reported improvements in motor function, gait speed, ADL, readmission, complications, and mortality with intensive RH, including increased delivery hours and frequency of interventions^11–13)^. However, most of these studies were conducted in post-acute care hospitals, with few reports in acute care hospitals. Furthermore, the relationship between time to home discharge and RH provision volume remains unclear.

In Japan, which has the highest population aging rate in the world, analysis utilizing extensive databases on this disease, which is commonly seen in the elderly, is expected to provide useful insights for other countries that will also experience population aging in the future. The Diagnosis Procedure Combination (DPC) database used in our study is Japan’s most extensive medical database, with approximately 7 million inpatient data entries submitted annually from over 1000 medical institutions^11)^. This database covers approximately 92% of Japan’s tertiary emergency hospitals, enabling a detailed understanding of acute care inpatient treatment nationwide^12)^. Additionally, comprehensive information related to medical care—including diagnostic names, treatment content, surgeries, RH, discharge outcomes, and medical resource utilization—is systematically recorded in a standardized format, making immediate and highly accurate analysis possible, which is a significant feature of this database. Using this database, we investigated the impact and magnitude of the RH starting date and RH provision volume on time to home discharge in patients with hip fractures.

## Methods

### Design and data sources

This observational study used inpatient data from the DPC database of the Diagnostic Group Classification Research Support Organization in Japan^13)^. The DPC database contains administrative claim data and detailed clinical data, including the following information: (1) demographic information such as gender, age, and admission route; (2) medical information related to diagnosis; (3) medical procedure information regarding procedures performed during hospitalization. The DPC database is widely used in medical and health research^13)^. Informed consent was waived. This study was approved by the Tohoku University Ethics Committee (Approval Number: 2021-1-1082).

### Patient selection

We used inpatient data from April 2020 to March 2022. Data were treated as 1 case per hip joint. Eligible patients were those who were hospitalized with an emergency for a hip fracture and who underwent only 1 operation for a hip fracture during 1 hospitalization. Hip fractures were defined as femoral neck fracture (S7200), femoral condyle fracture (S7210), or subtrochanteric femoral fracture (S7220) according to the International Classification of Diseases, 10th edition (ICD-10) codes. The exclusion criteria were those who met the following conditions: (1) patients under 65 years of age; (2) patients presenting with bilateral injuries; (3) patients with COVID-19 during hospitalization; (4) patients who have not undergone RH; (5) patients with surgical delay. For surgical delay, we followed a previous study^14)^ and defined surgery within 7 days of admission as having no surgical delay and surgery after that time as having a surgical delay.

### Variables

We determined variables to be extracted based on previous studies and existing knowledge on length of stay and home discharge, and the necessary data were collected from the DPC database. The variables used for analysis were as follows: age, sex^15–19)^, body mass index (BMI)^15–17,19)^, Charlson Comorbidity Index (CCI), past or current smoking status^15,19)^, surgical type^18)^, days from admission to surgery^19)^, Barthel Index (BI) at admission^19,20)^, nursing care level^16)^, Major Diagnostic Category (MDC) code 07 per year, MDC code 16 per year^19)^, anticoagulants^21)^, antiplatelet agents^22)^, general anesthesia^23)^, epidural anesthesia, red blood cell (RBC) transfusion^19)^, intensive care unit or high care unit (ICU/HCU) admission, hemodialysis (HD)^17)^, dementia^24)^, pneumonia, pulmonary embolism (PE), deep vein thrombosis (DVT)^25)^, and preoperative RH^26)^.

For age, we used the Japan Geriatrics Society criteria^23)^ to categorize the patients into 3 age groups: 65–74 years, 75–89 years, and 90 years and older. Based on a previous study, BMI was classified into 3 categories^11)^: 18.4 kg/m^2^ or less, 18.5–29.9 kg/m^2^, and 30.0 kg/m^2^ or more. For BI at admission, we classified the non-independent group as having less than 60 points and the independent group as having 60 points or more, in line with previous studies^27,28)^. Past or current smoking status refers to the smoking index and categorizes patients as smokers or nonsmokers. Smokers include both those who are currently smoking and those who have smoked in the past. CCI was classified into 4 categories: low (0 points), medium (1–2 points), high (3–4 points), and very high (5 or more points), based on a previous study^29)^. RBC transfusions were categorized into those who received RBC transfusions by the third postoperative day and those who did not. The surgical type was classified by the surgical core code: open reduction and internal fixation, intra-articular fracture surgery, bipolar hip arthroplasty, and total hip arthroplasty. The nursing care level is a care indicator unique to Japan and is broadly classified into support required and nursing care required. The 7 categories are support 1, support 2, nursing care 1, nursing care 2, nursing care 3, nursing care 4, and nursing care 5, depending on the level of care required^30)^. The lightest level of care required is support 1, and the heaviest is nursing care 5. In this study, 8 categories were used, including none. We used the total number of discharges per year per MDC to indicate the size of facilities. MDC is based on the ICD-10 classification established by the World Health Organization and includes 18 major diagnostic groups, such as neurological, ophthalmologic, and otorhinolaryngological diseases. MDC07 refers to the number of cases of musculoskeletal disorders; MDC16 refers to the number of cases of trauma, burns, and poisoning disorders. Continuous variables such as MDC07 and MDC16 were classified into quartile categories based on previous studies^31)^. The days from admission to surgery were calculated using the day of admission as Day 0^32)^. HD was classified according to whether hemodialysis was performed or not. Dementia was classified based on whether or not patients had dementia (ICD-10: F00$–F03$) as a comorbid condition. Anticoagulants were classified according to whether or not oral anticoagulants (edoxaban, rivaroxaban, apixaban, warfarin) or parenteral anticoagulants (heparin, fondaparinux sodium, enoxaparin sodium) were used during hospitalization. Antiplatelet agents were classified according to whether or not cilostazol, clopidogrel, or aspirin were used during hospitalization. General anesthesia, epidural anesthesia, and ICU/HCU admission were classified according to their implementation. Pneumonia, PE, and DVT were classified based on whether they occurred during hospitalization. Preoperative RH was classified based on whether at least 1 unit of RH was performed by a physical or occupational therapist between the date of admission and the day before surgery.

### Evaluation of RH

In Japan, the DPC database records the amount of RH provided to each patient daily by the physical or occupational therapists. Each unit of RH lasted 20 min, and patients could receive up to 9 units. We chose 2 main exposure variables: the RH starting date and the RH provision volume. For the RH starting date, we surveyed the first postoperative day on which RH was performed by a physical or occupational therapist. Based on previous reports^33,34)^, we classified patients into groups that started RH on Day 0, Day 1, postoperative day 2 (Day 2), or postoperative day 3 or later (≥Day 3). For RH provision volume, following a previous study^35)^, we calculated the average number of units per day based on the total number of RH units during the first postoperative week. According to the calculated average number of daily units, we categorized our patients into 0–1.0, 1.1–2.0, 2.1–3.0 , 3.1–4.0, 4.1–5.0, and 5.1 units/day or higher.

### Outcomes

We defined outcome as time to home discharge. Home discharge was defined as the occurrence of an event, and the event period was defined as the period from Day 0 to home discharge. Patients who were not discharged home (e.g., transferred to another ward, long-term care/RH hospital, or clinic; admitted to a geriatric health service facility, elderly welfare facility, social welfare facility, or paid nursing home; or who died) were treated as censored^20)^.

### Statistical analysis

We presented each variable as number and percentage to examine the characteristics of RH starting date and RH provision volume. Next, we performed a Cox proportional hazards regression analysis to investigate the associations between time to home discharge, RH starting date, and RH provision volume. The proportionality of the hazard over time was visually confirmed by log [log (cumulative incidence of home discharge)] versus log (length of postoperative stay) log plots. We treated competing risks of death as censored at the date of death. A hazard ratio greater than 1 indicates that the speed of home discharge is faster and that home discharge is easier. On the other hand, a hazard ratio less than 1 indicates that home discharge takes longer and is more difficult^31)^. We obtained a correlation matrix for each factor to assess multicollinearity and excluded variables with correlation coefficients greater than 0.9. Statistical analysis was performed using JMP Pro version 17.0.0 (SAS Institute, Cary, NC, USA). The tests were 2-tailed and considered statistically significant when p <0.05.

### Sensitivity analysis

We performed the following 3 sensitivity analyses to demonstrate the robustness of our results. First, patients who underwent surgery within 6 days of admission were reanalyzed for our study. Second, patients who underwent surgery within 8 days of admission were reanalyzed for our research. Third, to address the possibility of selection bias arising from the exclusion of patients who did not undergo RH, those who did not undergo RH were also included in the target population and reanalyzed.

### Subgroup analysis

Next, we performed a subgroup analysis to verify whether the impact of the RH starting date and RH provision volume on the time to home discharge differed between patients with dementia and those without dementia.

## Results

**Fig. 1.**
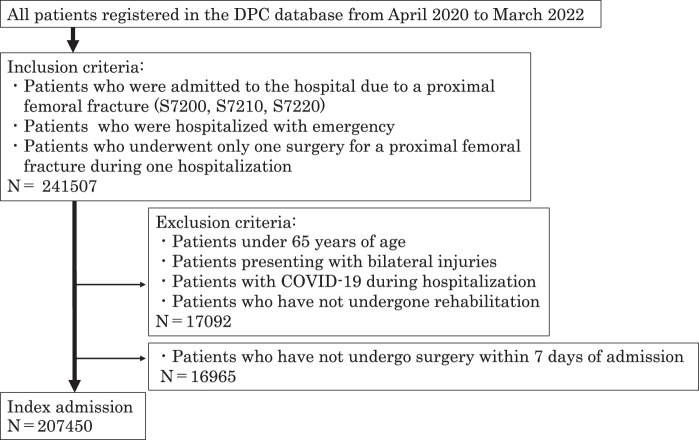
Patient selection flow. Delayed surgery was defined as a delay of 8 days or more between admission and surgery. DPC, Diagnosis Procedure Combination; COVID-19, Coronavirus disease 2019

Figure 1 shows patient selection. Of the 241507 patients who met the inclusion criteria, 34057 patients who met the exclusion criteria were excluded, resulting in 207450 patients for this study.

Table 1 shows the characteristics of the RH starting date. The RH starting date tended to be earlier in hospitals with more MDC16 cases, in patients with longer days before surgery, and in those who had received preoperative RH. On the other hand, the RH starting date tended to be later in patients who had received blood transfusions or hemodialysis and in those who stayed in the ICU.

**Table 1.** Relationship between patient characteristics and RH starting date

	RH started on Day 0 (N = 4532)	RH started on Day 1 (N = 162992)	RH started on Day 2 (N = 19046)	RH started on ≥Day 3 (N = 20880)
Days from admission to surgery, n (%)				
Day 0	295 (6.51)	17967 (11.02)	2828 (14.85)	3429 (16.42)
Day 1	1985 (42.80)	41208 (25.28)	4858 (25.51)	5667 (27.14)
Day 2	839 (18.51)	31867 (19.55)	3441 (18.07)	4114 (19.70)
Day 3	569 (12.56)	25638 (15.73)	2642 (13.87)	2872 (13.75)
Day 4	319 (7.04)	18344 (11.25)	2065(10.84)	2016 (9.66)
Day 5	222 (4.90)	12855 (7.89)	1494 (7.84)	1043 (5.00)
Day 6	166 (3.66)	9225 (5.66)	979 (5.14)	932 (4.46)
Day 7	137 (3.02)	5888 (3.61)	739 (3.88)	807 (3.86)
Preoperative RH, n (%)	1684 (37.16)	45769 (28.08)	2836 (14.89)	2479 (11.87)
Age, n (%)				
65–74	571 (12.60)	20231 (12.41)	2375 (12.47)	2603 (12.47)
75–89	2579 (56.91)	94080 (57.72)	10935 (57.41)	11903 (57.01)
≥90	1382 (30.49)	48681 (29.87)	5736 (30.12)	6374 (30.53)
Sex, female, n (%)	3601 (79.46)	127860 (78.45)	14871 (78.08)	16205 (77.61)
BMI, n (%)				
≤18.4	1180 (27.60)	43680 (28.62)	5060 (28.68)	5460 (28.56)
18.5–29.0	3026 (70.78)	106859 (70.02)	12350 (70.00)	13374 (69.95)
≥30	69 (1.61)	2076 (1.36)	233 (1.32)	285 (1.49)
CCI, n (%)				
Low	1481 (32.68)	59172 (36.30)	6842 (35.92)	7214 (34.55)
Medium	2341 (51.65)	82613 (50.69)	9413 (49.42)	10542 (50.49)
High	574 (12.67)	17675 (10.84)	2285 (12.00)	2591 (12.41)
Very high	136 (3.00)	3532 (2.17)	506 (2.66)	533 (2.55)
Nursing care level, n (%)				
None	1708 (38.78)	64398 (40.62)	7443 (40.33)	8046 (39.75)
Support required 1	199 (4.52)	7584 (4.78)	861 (4.66)	888 (4.39)
Support required 2	281 (6.38)	9226 (5.82)	1144 (6.20)	1186 (5.86)
Care level 1	624 (14.17)	21272 (13.42)	2407 (13.04)	2701 (13.34)
Care level 2	648 (14.71)	22725 (14.33)	2684 (14.54)	3111 (15.37)
Care level 3	547 (12.42)	19539 (12.33)	2258 (12.23)	2511(12.41)
Care level 4	302 (6.86)	10705 (6.75)	1292 (7.00)	1376 (6.80)
Care level 5	95 (2.16)	3081 (1.94)	368 (1.99)	422 (2.08)
Number of MDC07, n (%)				
≤346	1213 (26.77)	56836 (34.87)	5822 (30.57)	6494 (31.10)
347–618	1269 (28.00)	43040 (26.41)	4510 (23.68)	5248 (25.13)
619–991	1054 (23.26)	33485 (20.54)	4448 (23.35)	4685 (22.44)
≥992	996 (21.98)	29613 (18.18)	4266 (22.40)	4453 (21.33)
Number of MDC16, n (%)				
≤729	1093 (37.36)	64344 (39.48)	7686 (40.35)	9529 (45.64)
730–1028	1095 (24.16)	37456 (22.98)	4312 (22.64)	5006 (23.98)
1029–1378	800 (17.65)	30727 (18.85)	3665 (19.24)	3436 (16.46)
≥1379	944 (20.83)	30465 (18.69)	3383 (17.76)	2909 (13.93)
Past or current smoking status, n (%)	489 (11.97)	16518 (11.32)	1893 (11.08)	2337 (12.38)
RBC transfusion, n (%)	1583 (34.93)	54783 (33.61)	7716 (40.51)	7884 (37.76)
ICU/HCU admission, n (%)	132 (2.91)	6737 (4.13)	928 (4.87)	962 (4.61)
HD, n (%)	102 (2.25)	3073 (1.89)	796 (4.18)	708 (3.39)
General anesthesia, n (%)	3069 (67.72)	104052 (63.84)	11560 (60.70)	12660 (60.63)
Epidural anesthesia, n (%)	1550 (43.65)	60937 (46.97)	7671 (49.47)	8397 (49.74)
Dementia, n (%)	1185 (26.15)	36837 (22.60)	4178 (21.94)	4849 (23.22)
Surgical method, n (%)				
ORIF	2883 (63.61)	98071 (60.17)	11717 (61.52)	12966 (62.10)
Osteosynthesis	73 (1.61)	2496 (1.53)	337 (1.77)	352 (1.69)
BHA	1487 (32.81)	59907 (36.75)	6739 (35.38)	7312 (35.02)
THA	89 (1.96)	2518 (1.54)	253 (1.33)	250 (1.20)
BI at admission, ≥60 points, n (%)	323 (8.07)	13882 (9.81)	1791 (10.80)	1912 (10.50)
Anticoagulant drugs, n (%)	2236 (49.34)	72520 (44.49)	8339 (43.78)	8779 (42.05)
Antiplatelet drugs, n (%)	790 (17.43)	29233 (17.94)	3500 (18.38)	3817 (18.28)
Pneumonia, n (%)	123 (2.71)	4199 (2.58)	553 (2.90)	613 (2.94)
Deep vein thrombosis, n (%)	9 (0.20)	518 (0.32)	55 (0.29)	70 (0.34)
Pulmonary embolism, n (%)	397 (8.76)	7097 (4.35)	804 (4.22)	820 (3.93)

RH, rehabilitation; N/n, number; BMI, body mass index; CCI, Charlson Comorbidity Index; MDC, major diagnostic category; RBC, red blood cell; ICU/HCU, intensive care unit/high care unit; HD, hemodialysis; ORIF, open reduction and internal fixation; BHA, bipolar hip arthroplasty; THA, total hip arthroplasty; BI, Barthel Index

Table 2 shows the characteristics of the RH provision volume. The implementation of RH provision volume tended to be higher in hospitals with more MDC16 cases, patients who received general anesthesia, patients who required less care, and patients who underwent preoperative RH. On the other hand, the implementation of RH provision volume tended to be lower in patients who received blood transfusions, patients who received epidural anesthesia, patients with dementia, and patients who had a shorter time to surgery.

**Table 2.** Relationship between patient characteristics and RH provision volume

	0–1.0 units/day (N = 50100)	1.1–2.0 units/day (N = 85048)	2.1–3.0 units/day (N = 38857)	3.1–4.0 units/day (N = 19421)	4.1–5.0 units/day (N = 7830)	≥5.1 units/day (N = 6194)
Days from admission to surgery, n (%)						
Day 0	7417 (14.80)	9963 (11.71)	4014 (10.33)	1925 (9.91)	726 (9.27)	474 (7.65)
Day 1	13559 (27.06)	21459 (25.23)	9940 (25.58)	5186 (26.70)	2061 (26.32)	1513 (24.43)
Day 2	9512 (18.99)	16466 (19.36)	7616 (19.60)	3851 (19.83)	1590 (20.31)	1226 (19.79)
Day 3	7287 (14.54)	13073 (15.37)	6099 (15.70)	3041 (15.66)	1222 (15.61)	999 (16.13)
Day 4	5069 (10.12)	9525 (11.20)	4368 (11.24)	2174 (11.19)	861 (11.00)	747 (12.06)
Day 5	3356 (6.70)	6653 (7.82)	3031 (7.80)	1435 (7.39)	585 (7.47)	554 (8.94)
Day 6	2320 (4.63)	4806 (5.65)	2248 (5.79)	1075 (5.54)	469 (5.99)	384 (6.20)
Day 7	1580 (3.15)	3103 (3.65)	1541 (3.97)	734 (3.78)	316 (4.04)	297 (4.79)
Preoperative RH, n (%)	6046 (12.07)	19741 (23.21)	12306 (31.67)	7224 (37.20)	3767 (48.11)	3684 (59.48)
Age, n (%)						
65–74	5217 (10.41)	11134 (13.09)	5046 (12.99)	2506 (12.90)	1058 (13.51)	819 (13.22)
75–89	27781 (55.45)	49274 (57.94)	22704 (58.43)	11412 (58.76)	4563 (58.28)	3763 (60.75)
≥90	17102 (34.14)	24640 (28.97)	11107 (28.58)	5503 (28.34)	2209 (28.21)	1612 (26.03)
Sex, female, n (%)	38909 (77.66)	66295 (77.95)	30604 (78.76)	15548 (80.06)	6215 (79.37)	4966 (80.17)
BMI, n (%)						
≤18.4	13964 (30.65)	22415 (28.36)	10245 (27.86)	5095 (27.44)	2075 (27.28)	1586 (25.95)
18.5–29.0	31030 (68.11)	55538 (70.27)	25989 (70.67)	13208 (71.14)	5408 (71.10)	4436 (72.58)
≥30	567 (1.24)	1090 (1.37)	539 (1.47)	264 (1.42)	123 (1.629)	90 (1.47)
CCI, n (%)						
Low	16750 (33.43)	31226 (36.72)	14490 (37.29)	7171 (36.92)	2854 (36.45)	2218 (35.81)
Medium	26075 (52.05)	42445 (49.91)	19462 (50.09)	9844 (50.69)	3951 (50.46)	3132 (50.57)
High	5990 (11.96)	9430 (11.09)	4073 (10.48)	2035 (10.48)	866 (11.06)	731 (11.80)
Very high	1285 (2.56)	1947 (2.29)	832 (2.14)	371 (1.91)	159 (2.03)	113 (1.82)
Nursing care level, n (%)						
None	17016 (35.07)	35035 (42.44)	16280 (43.00)	7692 (40.60)	3056 (39.83)	2516 (41.34)
Support required 1	2010 (4.14)	3947 (4.78)	1868 (4.93)	1006 (5.31)	391 (5.10)	310 (5.09)
Support required 2	2683 (5.53)	4798 (5.81)	2295 (6.06)	1184 (6.25)	479 (6.24)	398 (6.54)
Care level 1	6633 (13.67)	10818 (13.11)	5020 (13.26)	2638 (13.93)	1005 (13.10)	890 (14.62)
Care level 2	7800 (16.08)	11548 (13.99)	5179 (13.68)	2664 (14.06)	1134 (14.78)	843 (13.85)
Care level 3	7052 (14.53)	9709 (11.76)	4326 (11.43)	2198 (11.60)	911 (11.87)	659 (10.83)
Care level 4	4018 (8.28)	5211 (6.31)	2271 (6.00)	1215 (6.41)	573 (7.47)	387 (6.36)
Care level 5	1309 (2.70)	1478 (1.79)	625 (1.65)	347 (1.83)	124 (1.62)	83 (1.36)
Number of MDC07, n (%)						
≤346	11965 (23.88)	26515 (31.18)	17153 (44.14)	8885 (45.75)	3432 (43.83)	2415 (38.99)
347–618	14182 (28.31)	24330 (28.61)	9290 (23.91)	3742 (19.27)	1537 (19.63)	986 (15.92)
619–991	12572 (25.09)	18410 (21.65)	6966 (17.93)	3257 (16.77)	1333 (17.02)	1134 (18.31)
≥992	11381 (22.72)	15793 (18.57)	5448 (14.02)	3537 (18.21)	1528(19.51)	1659 (26.78)
Number of MDC16, n (%)						
≤729	19299 (38.52)	34466 (40.53)	17090 (43.98)	7926 (40.81)	2898 (37.01)	1573 (25.40)
730–1028	12104 (24.16)	20838 (24.50)	8609 (22.16)	4122 (21.22)	1523 (19.45)	673 (10.87)
1029–1378	10012 (19.98)	15413 (18.12)	6763 (17.40)	3503 (18.04)	1245 (15.90)	1692 (27.32)
≥1379	8685 (17.34)	14331 (16.85)	6395 (16.46)	3870 (19.93)	2164 (27.64)	2256 (36.42)
Past or current smoking status, n (%)	5082 (11.28)	9113 (11.99)	3889 (11.22)	1831 (10.43)	746 (10.65)	576 (10.21)
RBC transfusion, n (%)	19584 (39.09)	29019 (34.12)	12721 (32.74)	6272 (32.29)	2581 (32.96)	1789 (28.88)
ICU/HCU admission, n (%)	2241 (4.47)	3657 (4.30)	1508 (3.88)	886 (4.56)	338 (4.32)	129 (2.08)
HD, n (%)	1732 (3.46)	1977 (2.32)	682 (1.76)	196 (1.01)	68 (0.87)	24 (0.39)
General anesthesia, n (%)	28274 (56.44)	53826 (63.29)	25478 (65.57)	13305 (68.51)	5675 (72.48)	4783 (77.22)
Epidural anesthesia, n (%)	22115 (53.95)	32043 (46.68)	14112 (45.64)	6501 (42.94)	2276 (39.21)	1508 (36.18)
Dementia, n (%)	12850 (25.65)	18768 (22.07)	8320 (21.41)	4276 (22.02)	1639 (20.93)	1196 (19.31)
Surgical method, n (%)						
ORIF	32417 (64.70)	51035 (60.01)	22984 (59.15)	11385 (58.62)	4416 (56.40)	3400 (54.89)
Osteosynthesis	809 (1.61)	1425 (1.68)	588 (1.51)	244 (1.26)	101 (1.29)	91 (1.47)
BHA	16363 (32.66)	31133 (36.61)	14637 (37.67)	7516 (38.70)	3212 (41.02)	2584 (41.72)
THA	511 (1.02)	1455 (1.71)	648 (1.67)	276 (1.42)	101 (1.29)	119 (1.92)
BI at admission, ≥60 points, n (%)	3987 (9.11)	8299 (11.36)	3394 (10.06)	1349 (7.84)	513 (7.38)	366 (6.50)
Anticoagulant drugs, n (%)	21142 (42.20)	38046 (44.73)	17251 (44.40)	8470 (43.61)	3781 (48.29)	3184 (51.40)
Antiplatelet drugs, n (%)	8978 (17.92)	15172 (17.84)	7060 (18.17)	3520 (18.12)	1494 (19.08)	1116 (18.02)
Pneumonia, n (%)	1681 (3.36)	2125 (2.50)	896 (2.31)	467 (2.40)	172 (2.20)	147 (2.37)
Deep vein thrombosis, n (%)	128 (0.26)	220 (0.26)	114 (0.29)	105 (0.54)	60 (0.77)	25 (0.40)
Pulmonary embolism, n (%)	1845 (3.68)	3666 (4.31)	1885 (4.85)	982 (5.06)	376 (4.80)	364 (5.88)

RH, rehabilitation; N/n, number; BMI, body mass index; CCI, Charlson Comorbidity Index; MDC, major diagnostic category; RBC, red blood cell; ICU/HCU, intensive care unit/high care unit; HD, hemodialysis; ORIF, open reduction and internal fixation; BHA, bipolar hip arthroplasty; THA, total hip arthroplasty; BI, Barthel Index

Table 3 presents the results of the Cox proportional hazards regression analysis. Both RH starting date and RH provision volume showed statistically significant associations. For the RH starting date, the adjusted hazard ratio based on RH started on ≥Day 3 was 0.97 (95% confidence interval: 0.93–1.02) for Day 2, 1.05 (1.01–1.09) for Day 1, and 1.18 (1.10–1.27) for Day 0. For RH provision volume, the adjusted hazard ratio based on the lowest dose (0–1.0 units/day) was 1.12 (1.08–1.15) for 1.1–2.0 units/day, 1.10 (1.06–1.14) for 2.1–3.0 units/day, 1.04 (1.00–1.08) for 3.1–4.0 units/day, 1.09 (1.04–1.14) for 4.1–5.0 units/day, and 1.11 (1.06–1.17) for ≥5.1 units/day. These showed proportional hazards characteristics.

**Table 3.** Cox proportional hazards regression analysis for time to home discharge

	Hazard ratio	95% CI	P value
Days from admission to surgery			
Day 0	Reference		
Day 1	0.96	0.93–1.00	0.0487
Day 2	0.90	0.87–0.94	<0.0001
Day 3	0.86	0.83–0.90	<0.0001
Day 4	0.83	0.80–0.87	<0.0001
Day 5	0.77	0.74–0.81	<0.0001
Day 6	0.77	0.73–0.81	<0.0001
Day 7	0.75	0.71–0.80	<0.0001
Preoperative RH	1.01	0.98–1.04	0.4633
RH starting date (days)			
≥Day 3	Reference		
Day 2	0.97	0.93–1.02	0.24
Day 1	1.05	1.01–1.09	0.009
Day 0	1.18	1.10–1.27	<0.0001
RH provision volume (units/day)			
0–1.0	Reference		
1.1–2.0	1.12	1.08–1.15	<0.0001
2.1–3.0	1.10	1.06–1.14	<0.0001
3.1–4.0	1.04	1.00–1.08	0.0328
4.1–5.0	1.09	1.04–1.14	0.0006
≥5.1	1.11	1.06–1.17	<0.0001
Age			
65–74	Reference		
75–89	0.64	0.62–0.65	<0.0001
≥90	0.55	0.53–0.57	<0.0001
Sex, female	1.15	1.12–1.18	<0.0001
BMI			
≤18.4	Reference		
18.5–29.0	1.01	0.99–1.04	0.2606
≥30	0.85	0.78–0.92	<0.0001
CCI			
Low	Reference		
Medium	0.94	0.92–0.96	<0.0001
High	0.87	0.84–0.90	<0.0001
Very high	0.84	0.79–0.89	<0.0001
Nursing care level			
None	Reference		
Support required 1	0.70	0.67–0.73	<0.0001
Support required 2	0.65	0.62–0.67	<0.0001
Care level 1	0.55	0.54–0.57	<0.0001
Care level 2	0.59	0.57–0.61	<0.0001
Care level 3	0.63	0.60–0.65	<0.0001
Care level 4	0.70	0.67–0.74	<0.0001
Care level 5	0.82	0.76–0.89	<0.0001
Number of MDC07 (cases)			
≤346	Reference		
347–618	1.18	1.15–1.21	<0.0001
619–991	1.29	1.26–1.34	<0.0001
≥992	1.43	1.38–1.48	<0.0001
Number of MDC16 (cases)			
≤729	Reference		
730–1028	1.03	1.00–1.06	0.0363
1,029–1378	0.9	0.87–0.92	<0.0001
≥1379	0.99	0.95–1.02	0.4957
Past or current smoking status	1.12	1.08–1.15	<0.0001
RBC transfusion	0.74	0.72–0.76	<0.0001
ICU/HCU admission	0.67	0.63–0.70	<0.0001
HD	0.81	0.76–0.87	<0.0001
General anesthesia	1.00	0.98–1.02	0.6709
Dementia	0.85	0.83–0.88	<0.0001
Surgical method			
ORIF	Reference		
Osteosynthesis	1.29	1.21–1.39	<0.0001
BHA	1.18	1.16–1.21	<0.0001
THA	1.97	1.86–2.09	<0.0001
BI at admission, ≥60 points	1.32	1.28–1.36	<0.0001
Anticoagulant drugs	0.93	0.92–0.95	<0.0001
Antiplatelet drugs	0.87	0.85–0.89	<0.0001
Pneumonia	0.35	0.33–0.38	<0.0001
Deep vein thrombosis	0.67	0.58–0.77	<0.0001
Pulmonary embolism	0.92	0.88–0.96	0.0003

Adjusted for age, sex, BMI, CCI, nursing care level, number of MDC07, number of MDC16, past or smoking status, RBC transfusion, ICU/HCU admission, HD, general anesthesia, dementia, surgical method, days until surgery, BI at admission, anticoagulants drugs, antiplatelet drugs, pneumonia, deep vein thrombosis, pulmonary embolism, and preoperative RH.

A hazard ratio greater than 1 indicates faster speed to home discharge (i.e., easier home discharge). A hazard ratio less than 1 indicates slower speed to home discharge (i.e., more difficult discharge).

CI, confidence interval; RH, rehabilitation; BMI, body mass index; CCI, Charlson Comorbidity Index; MDC, major diagnostic category; RBC, red blood cell; ICU/HCU, intensive care unit/high care unit; HD, hemodialysis; ORIF, open reduction and internal fixation; BHA, bipolar hip arthroplasty; THA, total hip arthroplasty; BI, Barthel Index

Supplementary Tables 1, 2, and 3 present the results of sensitivity analyses. In the 3 sensitivity analyses, the associations of RH starting date and RH provision volume with time to home discharge were similar to those in the primary analysis.

Supplementary Table 4 presents the results of subgroup analyses. In patients without a dementia diagnosis, the associations of RH starting date and RH provision volume with time to home discharge were similar to those in the primary analysis. On the other hand, the results for patients with dementia differed from those in the primary and sensitivity analyses.

In the correlation matrix for each covariate, “epidural anesthesia” was excluded from the variables because the correlation coefficient between “general anesthesia” and “epidural anesthesia” was greater than 0.9.

## Discussion

In our study, we used the Japanese DPC database to investigate the impact of the RH starting date and provision volume on the time to home discharge. The results showed that both factors were independently associated with the time to home discharge.

Our study has several strengths. First, due to the characteristics of the DPC database, the results are readily generalizable nationwide and provide valuable insights for developing new standards. Second, we used the time to home discharge as an outcome. Previous studies have focused on length of stay as an outcome^36)^; however, the discharge destination has not always been taken into consideration. Since the longer the length of stay, the higher the likelihood of home discharge, it is important to consider the time to discharge to verify the efficacy of home discharge. Our study can be considered a practical and novel analysis in that it focuses on the speed of home discharge. Third, we confirmed the robustness of the results by performing multiple sensitivity analyses. The results remained consistent even when the period from admission to surgery was varied. Fourth, we newly found that patients with lower nursing care levels tended to have a longer time to home discharge compared with those with higher nursing care levels. Generally, patients with higher nursing care levels already have an established home care infrastructure and well-coordinated support systems with family members and caregivers. Therefore, less time may be required to establish discharge support. Previous studies have also reported that the absence of home-based care services is a factor associated with delayed home discharge after hip fracture surgery^37)^.

Takahashi et al.^38)^ reported that delayed RH starting is associated with prolonged hospitalization. Our findings are consistent with this. Particularly, we found that starting RH on Day 0 contributes to increased home discharges and reduced length of stay. However, starting RH on Day 0 of hospitalization requires prior coordination with physicians and an organized effort across the entire facility.

In terms of the effects of RH provision volume, Uda et al.^32)^ examined the impact of the RH starting date, frequency (number of days per week), and daily provision volume on ADL outcomes after hip fracture surgery in patients with dementia. They reported a dose–response relationship between increased daily provision volume and ADL score at discharge. Our study suggested that providing at least 1 unit of RH may be associated with shorter time to home discharge, but there was no dose–response. Furthermore, we found no clear evidence on shortening the time to home discharge in patients with dementia in subgroup analyses.

There may be several potential factors underlying these results that could not be considered due to the characteristics of the DPC database^16,20,39,40)^: (1) environmental factors such as family structure, access to social support, and housing conditions; (2) factors such as the therapist’s skill level and proficiency, and differences based on clinical pathways established by each hospital; (3) physical and cognitive factors such as frailty, nutritional status, pain management or intensity, and severity of cognitive impairment; and (4) psychological factors such as motivation for RH and the presence of depressive symptoms.

There are also several other limitations to our study. First, the content of the RH (e.g., range of motion exercises and walking exercises) and the practitioners (physical therapists and occupational therapists) are unclear. Second, due to the limitations of the DPC database, it is not possible to track the clinical course of patients after they are transferred to other hospitals. These limitations should be taken into account when interpreting the results.

## Conclusions

Starting RH on Day 0 was suggested to potentially facilitate faster or easier home discharge. Although no clear dose–response relationship was observed between RH provision volume and time to home discharge, providing at least 1 unit of RH per day appeared to be effective.

## Supplementary Materials

Supplementary Table 1. Cox proportional hazards regression analysis of time to home discharge when surgical delay is defined as up to 6 days after admission.

Supplementary Table 2. Cox proportional hazards regression analysis of time to home discharge when surgical delay is defined as up to 8 days after admission.

Supplementary Table 3. Cox proportional hazards regression analysis of time to home discharge when patients not undergoing rehabilitation are included in this study.

Supplementary Table 4. Subgroup analysis of time to home discharge based on whether or not the patient has dementia.
