# Supplementary material for: Impact of Rehabilitation on Time to Home Discharge after Hip Fracture Surgery: A Retrospective Observational Study Using the Japanese Nationwide Database of Diagnosis Procedure Combination
Source: Phys Ther Res. 2026 Feb 20;29(1):31–41. doi: 10.1298/ptr.25-E10360 (PMC13143131; doi:10.1298/ptr.25-E10360)
Supplement: Supplementary file 2 — Supplementary Table 2. Cox proportional hazards regression analysis of time to home discharge when surgical delay is defined as up to 8 days after admission. [file ptr-29-31-s002.pdf]

		Hazard Ratio	95% CI	P value
Days from admission to surgery (days)	Day 0	Reference		
	Day 1	0.96	0.93-1.00	0.0468
	Day 2	0.90	0.87-0.94	<0.0001
	Day 3	0.86	0.83-0.90	<0.0001
	Day 4	0.83	0.80-0.87	<0.0001
	Day 5	0.77	0.74-0.81	<0.0001
	Day 6	0.77	0.73-0.81	<0.0001
	Day 7	0.75	0.71-0.80	<0.0001
	Day 8	0.76	0.71-0.80	<0.0001
Preoperative RH		1.01	0.98-1.03	0.4821
RH starting date (days)	≥Day 3	Reference		
	Day 2	0.98	0.93-1.02	0.3577
	Day 1	1.04	1.01-1.08	0.004
	Day 0	1.19	1.10-1.28	<0.0001
RH provision volume (units/day)	0–1.0	Reference		
	1.1–2.0	1.12	1.08-1.14	<0.0001
	2.1–3.0	1.10	1.06-1.13	<0.0001
	3.1–4.0	1.04	1.00-1.08	0.0417
	4.1–5.0	1.09	1.04-1.14	0.0006
	≥5.1	1.10	1.05-1.16	<0.0001
Age	65–74	Reference		
	75–89	0.64	0.62-0.65	<0.0001
	≥90	0.55	0.53-0.57	<0.0001
Sex, female		1.14	1.11-1.17	<0.0001
BMI	≤18.4	Reference		
	18.5–29.0	1.02	0.99-1.04	0.1385
	≥30	0.85	0.79-0.92	<0.0001
CCI	Low	Reference		
	Medium	0.94	0.92-0.96	<0.0001
	High	0.86	0.84-0.90	<0.0001
	Very High	0.84	0.79-0.90	<0.0001
Nursing care level	None	Reference		
	Support required1	0.70	0.67-0.73	<0.0001
	Support required2	0.65	0.62-0.67	<0.0001
	Care Level 1	0.55	0.54-0.57	<0.0001
	Care Level 2	0.59	0.57-0.61	<0.0001
	Care Level 3	0.63	0.60-0.65	<0.0001
	Care Level 4	0.70	0.67-0.73	<0.0001
	Care Level 5	0.81	0.75-0.88	<0.0001
Number of MDC07 (cases)	≤346	Reference		
	347–618	1.18	1.15-1.21	<0.0001
	619–991	1.29	1.25-1.33	<0.0001
	≥992	1.43	1.38-1.48	<0.0001
Number of MDC16 (cases)	≤729	Reference		
	730–1028	1.03	1.00-1.06	0.0201
	1029–1378	0.89	0.87-0.92	<0.0001
	≥1379	0.99	0.96-1.03	0.5997
Past or current smoking status		1.11	1.08-1.15	<0.0001
RBC transfusion		0.74	0.73-0.76	<0.0001
ICU/HCU admission		0.67	0.63-0.70	<0.0001
HD		0.81	0.75-0.86	<0.0001
General anesthesia		1.00	0.98-1.02	0.6805
Dementia		0.85	0.83-0.88	<0.0001
Surgical method	ORIF	Reference		
	Osteosynthesis	1.28	1.20-1.37	<0.0001
	BHA	1.18	1.16-1.21	<0.0001
	THA	1.98	1.87-2.10	<0.0001
BI at admission, ≥60points		1.32	1.28-1.36	<0.0001
Anticoagulant drugs		0.94	0.92-0.95	<0.0001
Antiplatelet drugs		0.87	0.85-0.89	<0.0001
Pneumonia		0.35	0.33-0.38	<0.0001
Deep Vein Thrombosis		0.66	0.57-0.76	<0.0001
Pulmonary Embolism		0.93	0.89-0.97	0.0006

Supplementary Table 2. Cox proportional hazards regression analysis of time to home discharge when surgical delay is defined as up to 8 days after admission.
Adjusted for Age, Sex, BMI, CCI, Nursing care level, Number of MDC07, Number of MDC16, Past or smoking status, RBC transfusion, ICU/HCU admission, HD, General anesthesia, Dementia, Surgical method, Days until surgery, BI at admission, Anticoagulants drugs, Antiplatelet drugs, Pneumonia, Deep Vein Thrombosis, Pulmonary Embolism, and Preoperative RH.
RH, rehabilitaion. CI, confidence interval. CCI, Charlson Comorbidity Index. MDC, Major Diagnostic Category. ICU/HCU, Intensive Care Unit / High Care Unit. RBC, red blood cell. HD, hemodialysis. ORIF, open reduction and internal fixation. BHA, Bipolar Hip Arthroplasty. THA, Total Hip Arthroplasty. BI, Barthel Index.
A hazard ratio greater than 1 indicates that the speed of home discharge is faster and that home discharge is easier. A hazard ratio less than 1 indicates that home discharge takes longer and is more difficult.
