# Supplementary material for: Impact of Rehabilitation on Time to Home Discharge after Hip Fracture Surgery: A Retrospective Observational Study Using the Japanese Nationwide Database of Diagnosis Procedure Combination
Source: Phys Ther Res. 2026 Feb 20;29(1):31–41. doi: 10.1298/ptr.25-E10360 (PMC13143131; doi:10.1298/ptr.25-E10360)
Supplement: Supplementary file 4 — Supplementary Table 4. Subgroup analysis of time to home discharge based on whether or not the patient has dementia. [file ptr-29-31-s004.pdf]

		Patients without dementia			Patients with dementia		
		Hazard Ratio	95% CI	P value	Hazard Ratio	95% CI	P value
RH starting date (days)	≥Day 3	Reference			Reference		
	Day 2	0.97	0.93-1.02	0.2366	1.03	0.92-1.16	0.5922
	Day 1	1.05	1.01-1.08	0.0126	1.05	0.96-1.15	0.3101
	Day 0	1.18	1.10-1.27	<0.0001	1.64	1.38-1.95	<0.0001
RH provision volume (units/day)	0–1.0	Reference			Reference		
	1.1–2.0	1.12	1.09-1.15	<0.0001	1.12	1.04-1.20	0.0017
	2.1–3.0	1.10	1.07-1.14	<0.0001	1.08	0.99-1.18	0.055
	3.1–4.0	1.05	1.01-1.09	0.0207	1.02	0.93-1.21	0.6637
	4.1–5.0	1.10	1.04-1.15	0.0002	1.06	0.93-1.21	0.3791
	≥5.1	1.12	1.07-1.18	<0.0001	1.14	0.98-1.33	0.0819

Supplementary Table 4. Subgroup analysis of time to home discharge based on whether or not the patient has dementia.
Adjusted for Age, Sex, BMI, CCI, Nursing care level, Number of MDC07, Number of MDC16, Past or smoking status, RBC transfusion, ICU/HCU admission, HD, General anesthesia, Surgical method, Days until surgery, BI at admission, Anticoagulants drugs, Antiplatelet drugs, Pneumonia, Deep Vein Thrombosis, Pulmonary Embolism, and Preoperative RH.
RH, rehabilitaion. CI, confidence interval. CCI, Charlson Comorbidity Index. MDC, Major Diagnostic Category. ICU/HCU, Intensive Care Unit / High Care Unit. RBC, red blood cell. HD, hemodialysis. ORIF, open reduction and internal fixation. BHA, Bipolar Hip Arthroplasty. THA, Total Hip Arthroplasty. BI, Barthel Index.
A hazard ratio greater than 1 indicates that the speed of home discharge is faster and that home discharge is easier. A hazard ratio less than 1 indicates that home discharge takes longer and is more difficult.
